# Feeling stretched: Parents’ narratives about challenges to resilience when their child has a tracheostomy

**DOI:** 10.1177/13674935231169409

**Published:** 2023-04-12

**Authors:** Alison Flynn, Karen Whittaker, Adam J Donne, Lucy Bray, Bernie Carter

**Affiliations:** 1 4593 Alder Hey Children’s NHS Foundation Trust Liverpool, Merseyside, UK; 2 Faculty of Health and Wellbeing, University of Central Lancashire, Preston, UK; 3 Faculty of Health, Social Care and Medicine, 6249 Edge Hill University, Ormskirk, Lancashire, UK

**Keywords:** tracheostomy, resilience, parent, children, narrative

## Abstract

This study aimed to examine how parents develop personal resilience when facing the challenges of caring for a child with tracheostomy. This study employed a longitudinal qualitative design. Unstructured narrative interviews with 12 parents (from nine families) whose child had a new tracheostomy were undertaken at three time points over 12 months. Data were analysed using a socio-narratology method. Findings reveal the journey parents experienced, how their feelings changed and the processes involved in developing resilience over the first 12 months of their child having a tracheostomy. Stories told by parents early in their journey revealed emotional upheaval, negative emotions, stress and shock. Due to medical need, parents had little or no choice for their child to have a tracheostomy. Once their child’s life was out of danger, parents started to reframe their experiences and beliefs. Resilience played a major part in how parents perceived and faced their situation, allowing them to deal with what came their way and to move forward with their lives. Different aspects of resilience such as self-awareness, grit, gratitude, internal locus of control and reframing came to the fore at different time points. Parents talked feeling stretched by the challenges they faced and how they reframed their perspectives about their child’s tracheostomy. Parents’ resilience and reframing is discussed in relation to the ABC-X model. This study identifies a theoretical model that explains this process of change, this results in transferable knowledge, useful for understanding and explaining the experience of other parents and families.

## Introduction

Tracheostomy in children is undertaken to create a safe airway for effective respiration when either there is an airway obstruction and/or neurological impairment causing respiratory dysfunction (Watters, 2017). Children with airway obstruction and neurological conditions require significant resource including multi-disciplinary team care and services to support them to be discharged from hospital and live at home with their families (Callans et al., 2016).

Only a few studies address the care experiences of parents whose child has a tracheostomy; thus, the evidence base is limited (Flynn et al., 2013). Two studies have reported parents’ psychological burden when caring for their child with a tracheostomy, such as feeling stressed about their child’s safety and constant worry about their own ability to manage (Harnick et al., 2003; Montagnino and Maurio, 2004). McNamara et al. (2009) present the core concept of ‘living worried’ and the constant uncertainty for parents of a child who has a tracheostomy. ‘Living worried’ included disrupted sleep and exhaustion due to managing their child’s care. Further evidence on parents’ experiences of caring for their child with complex needs suggests they face daily struggles given the emotionally demanding nature of the medical procedures they are required to undertake (McDonald et al., 2016) and on-going sleep deprivation can lead to poor mental health and relationship difficulties (McCann et al., 2015). The studies specific to children with a tracheostomy focus on challenges and burdens but none of them have addressed the development of coping strategies or resilience as concepts or their potential influence on or benefits for parents whose child has had a tracheostomy.

Resilience is a complex construct that has been clouded by a lack of consensus in terms of definition and processes (Windle, 2011) and by conceptual ambiguity (Ungar 2019). A long-standing definition proposes that the principal features of resilience tend to include an ability to hold up to stress and withstand being stretched but also being able to spring back and achieve a positive outcome (Masten, 2001). More contemporary work aiming to achieve consensus on defining resilience showed agreement on ‘conceptualizing resilience at multiple levels, from the biological to the social structural level, a focus on the dynamic nature of resilience, and a move away from conceptualizing resilience as only an individual trait’ (Denckla et al., 2020: 1). Being resilient does not necessarily mean that an individual is unaffected or untouched by adversity or that an individual faced with adversity will always function well (Southwick et al., 2014). Having a child with a tracheostomy represents a major life changing event for the entire family and resilience could be a key concept in how parents adjust to new challenges. Resilience research has demonstrated the potential to support the empowerment of and stability of families (Noyes, 2014). Most family-focussed, theoretical models of resilience (Ellingsen et al., 2014; McCubbin and Patterson, 1983; Patterson, 1988) in contemporary use build on the ABC-X model of family stress (Hill, 1949) which aimed to investigate how families adapted and adjusted to stressful and complex situations. Ellingsen et al.’s (2014) version of the ABC-X model explains how risk factors can be buffered by protective factors to achieve an outcome of interest. This model did not a priori inform the study design but was used to frame the findings and discussion in this paper.

This paper is drawn from work from a PhD programme of work (Flynn, 2018).

## Aim

To examine how parents develop personal resilience when facing the challenges of caring for a child with tracheostomy over the initial 12 months.

## Method

Narrative inquiry (Riessman, 2008) was adopted as the methodological approach for this longitudinal interview-based study conducted at three time points over 12 months. The time points (before discharge, after three months at home and 12 months after tracheostomy was performed) were based on clinical insight into experiences of families’ journeys and how they progress from hospital to home. Ethics approval was gained from the University ethics committee (BuSH 151) and National Research Ethics Service (NRES 13/NW/0349). A reflexive approach was adopted throughout the qualitative study to manage potential impacts from the nurse-researcher role on recruitment, data collection and analysis arising. The lead researcher (AF) had a clinical relationship with all participants, and therefore made purposeful efforts to reduce the power differential with parents by discussing and explaining the difference between the research and clinical roles. Care was taken to ensure that the participants led the narrative interviews, creating opportunities for them to raise issues of importance to them, hence reducing researcher influence on the generation of data. To limit any ‘clinical lens’ bias from the lead researcher, the analysis process also involved members of the team who were less familiar with clinical aspects of tracheostomy care.

### Sampling and recruitment

Convenience sampling was the pragmatic choice of sampling method. Convenience sampling in this study involved the identification and approach of all parents who met the inclusion criteria and were available at the one study site during the recruitment period. The inclusion criteria were parents, main carers and foster carers of a child (aged from birth − 16 years) who had a new tracheostomy; and were attending the study hospital (a specialist children’s hospital in the North-West of England). Parents who could not sustain a conversation in English were excluded from participation as narrative inquiry depends on the researcher’s interpretation of stories.

The recruitment process used a two-stage approach. Stage 1: the child’s Ear, Nose and Throat (ENT) consultant approached potential participants 1–3 weeks following insertion of their child’s tracheostomy and briefly informed them about the study and gave them a written information leaflet. Stage 2: if potential participants demonstrated an interest in the study the lead researcher (AF) approached them at a suitable time for parents and provided a more in-depth verbal explanation, plus written information and answered any questions. If the parent(s) wished to participate, informed written consent was then gained.

### Narrative interview

The primary method for narrative research is the unstructured interview (Chauhan, 2019). In this approach, participants are not restricted or guided by the researcher’s agenda and participants can control the direction and content of the interview (Ziebland, 2013). The face-to-face interviews were undertaken by the lead researcher (AF). The opening questions for each interview were as follows: time point 1 (before discharge from hospital), ‘(Child’s name) has had a tracheostomy, please tell me about this experience’; time point 2 (3 months post discharge), ‘How are you getting on?’; and time point 3 (12 months post discharge), ‘Looking back now, how are you feeling and looking forward now, how do you see the future?’ The average interview time was 90 min. Some parents became upset during the interview, at this point, they were asked if they would like to pause the interview, take a break, stop or to continue. Parents were debriefed after their interview, offered a sheet providing details of support organisations and including a link to the psychology service within the recruiting hospital if needed. No parents sought support via the hospital.

In the interviews, prompts were used, as needed, to help the parent’s story unfold. The interviews were undertaken either within the hospital setting or the family home, depending on the time point or family preference. All the interviews were audio-recorded.

### Data analysis

The audio-files were transcribed and anonymised. A socio-narratology approach (Frank, 2010) was chosen to gain deep understanding and insight into the parents’ experiences. The socio-narratology approach draws the attention of the researcher to the work that stories have the ‘capacity to do’ (Frank, 2010: 4), and the researcher applies questions from story-telling practice (e.g. ‘What makes a story narratable’? and ‘Who is holding their own’?) to the transcripts. Using these questions allowed for a movement of thought within the data and a robust insight into the experiences parents shared. The focus of this method is on what is voiced in a story, the subject of the story, and the effects of telling a story (Frank, 2010). As analysis was coming toward completion, it became clear that the themes strongly clustered around the concept of resilience. A deep dive into the resilience literature resulted in the decision for the final round of analysis to be informed by Ellingsen et al.’s (2014) ABC-X model as the core elements helped to present the parents’ stories.

Pseudonyms were chosen for the children to protect their anonymity and that of their parents. Quotations are used to illustrate the text, and labels are used to indicate the participants: child’s pseudonym, followed by their mother/father’s name, as appropriate.

## Findings

### Demographics

Twenty-three narrative interviews were undertaken with 12 parents (three fathers and nine mothers) from nine families (see Table 1). Matthew’s mother withdrew from the study after the first interview as she said she found it hard to talk about their experiences and be audio-recorded. Anna’s parents withdrew due to family issues. Both parents did not withdraw their data from the study.

**Table 1.** Family demographics.

Family composition (including child’s pseudonym)	Child’s age at time of tracheostomy	Child’s principal diagnoses	Who was interviewed and number of interviews undertaken
**Tom**	6 weeks old	Di george syndrome	Mother and father
His mother and father and one sibling			Three interviews
**Rose**	4 months	Small airway due to complex medical issues	Mother
Her mother and father and one sibling			Three interviews
**Sam**	2 years	Complex neuro disability	Mother and father
His mother and father			Three interviews
**Melody**	3 months	Airway obstruction due to congenital abnormalities	Mother
Her mother and father			Three interviews
**Matthew**	4 weeks old	Laryngeal cleft	Mother
His mother and two siblings			One interview
**Freya**	3 months	Vocal cord paralysis	Mother
Her mother and father and one sibling			Three interviews
**Louise**	11 years old	Complex neuro disability	Mother
Her mother and father and one sibling			Three interviews
**Jack**	9 months	Subglottic stenosis	Mother
His mother and father and one sibling			Three interviews
**Anna**	4 weeks old	Pierre robin syndrome	Mother and father
Her mother and father			One interview

Resilience, to a greater or lesser degree, was evident across all the parents’ stories. Parents reported being able to manage adversity and reflect on and develop a resilient mind-set over time about their child’s tracheostomy and health care needs. Parents reflected and recognised that there were times when they exhibited higher levels of resilience related to caring for their child and times when their resilience was lower. Parents talked about being constantly challenged and feeling stretched by the demands associated with caring for their child; these challenges changed over 12 months as they developed resilience. Resilience themes that emerged from the data such as self-awareness, grit, gratitude, internal locus of control and reframing came to the fore at different time points (see Table 2). The three time points are used to structure the findings.

**Table 2.** Overview of parents’ key feelings and aspects of resilience.

Time frame	Typical feelings experienced by parents	Key aspects of resilience developed by parents
The first 3 months (hospitalisation, investigations, creation of a tracheostomy, developing skills)	Negative emotions (shock, feeling stretched, scared, overwhelmed)	Self-awareness
		Grit
Months 4 to 6 (going home, managing initial challenges of being home)	Readjustment and growth (feeling stretched at times, relief, support)	Gratitude
		Internal locus of control
Months 7–12 (sustaining care at home, coming out the other side)	Feeling more positive (reflection, hope)	Reframing

### The first 3 months: Negative emotions and developing self-awareness and grit

The early experiences for parents of their child being ill were stressful, complex and complicated and were often associated with negative reactions and emotions towards their child needing a tracheostomy. The need for their child to have a tracheostomy challenged parents and they framed this as something over which they had little control. Parents described how stretched their life felt as struggling to make decisions about their child’s surgery was often difficult, *‘I was in shock, I was so upset’* (Matthew’s mother). Parents reported their emotions as being stretched to the limit:

‘I was telling them, she needs to go to specialist hospital, she can’t breathe’ (Anna’s mother).

The parents talked of how scared they were when finding out that their child had life threatening breathing issues. Parents described feeling that it was unfair that their child needed a tracheostomy and perceived this to be the ‘*end of the world’* (Sam’s father) with one father reporting having a ‘*breakdown’* as a result. Parents described initially feeling that their child needing a tracheostomy was worse than other diagnoses, with Tom’s father saying it is ‘*worse than having a mental issue’.*

Reflecting on their emotions, as parents became self-aware about their negative feelings towards the tracheostomy, such as how the tracheostomy relieved their child’s suffering. After watching his son suffer with his breathing, modified his negative feelings Tom’s father explained:

‘you got to cope with it, it needs to be done, he is your child’ (Tom’s father).

All parents openly discussed their reservations associated with their child needing a tracheostomy. The key reservation was the stigma they associated with a tracheostomy. However, when the tracheostomy relieved their child’s breathing problem, they reflected that their initial negative feelings had been *‘misplaced’* (Sam’s father).

Despite having undertaken the tracheostomy training programme provided by the hospital, all the parents reported the constant worry about their ability to manage their child’s tracheostomy care successfully. Parents found it tough when changing their child’s tracheostomy tube for the first time, as this required them to overcome their anxiety about performing a task their child’s life depended on. Anna’s father described how he felt ‘*horrible, but I got to do it right for her’* and Sam’s mother talked of how incredibly worried she was about the first tube change for her son, remembering thinking: *‘I am not going to be able to do it, but then I knew I had to do it’* (Sam’s mother). Anna’s mother recalled her anxiety and emotions and indicated the grit needed to hold things together:

‘[I was] dead anxious… I was thinking what if it [tube] doesn’t go in. But it does and you do do it. It just happens doesn’t it? But ….after I had done the trachy change, that’s when I panicked. I felt me body, you know tears in my eyes and stuff ‘cause in a way, I was happy that I had done it. I was so anxious before and it was very emotional, …it’s a scary thing’ (Anna’s mother).

The parents demonstrated high levels of grit, determination and resilience in such situations and these resources helped them face their fears.

### Months 4 to 6: Readjustment and growth, developing gratitude and gaining an internal locus of control

In months 4 to 6, parents experienced times of readjustment, growth and further challenges. Despite having gained skills and become used to their child’s care, preparing to go home and the prospect of facing other people’s reactions to their child’s tracheostomy renewed their anxieties and uncertainty. Parents described feeling *‘full of nerves’* (Tom’s mother) and ‘*worrying*’ (Louise’s mother) about their ability to cope at home.

Parents talked about their worry and fears of venturing outside of their homes; Jack’s mother perceived some places as being off limits. Parents also recalled the times when they had to deal with other people’s reactions to their child’s tracheostomy. Melody’s mother recalled times when she was in a supermarket and Melody needed suctioning and had been challenged people who objected to her doing this. She described her response:

‘I just turned around and said, “Well do you prefer a dead baby next to you instead?”…… They said “How dare you do that it’s disgusting, why do you do that in front of people?”. You just feel like….[it’s] obviously something wrong and they don’t know how hard [Melody] had to literally fight to even be alive, so it just annoys me more than anything, and I feel that they are too rude’ (Melody’s mother).

However, during this period, expressing a sense of gratitude that the tracheostomy had saved their child’s life became a key feature of conversations and reflecting on this mitigated parents’ negative feelings. Feeling gratitude such as ‘*we’re really really grateful for it [tracheostomy]’* (Tom’s father), created a sense of well-being for parents. Parents valued the gift of their child being alive:

‘So what, she got a trachy, she is here, she’s alive’ (Rose’s mother).

This brought a perspective which allowed them to move forward towards a more certain future as a family.

Once parents had acquired the clinical skills and confidence to care for their child, an overall sense of control came back to their everyday lives. The parents developed a strong internal locus of control which helped them, as individuals, to buffer the effects of their negative experiences and the external factors that were out of their control such as their child needing a tracheostomy. One father discussed how he managed his son’s tracheostomy care with confidence, saying ‘*It’s old hat now the trachy, it’s nothing, you become use to it*’ (Sam’s father). Parents took control emotionally, with one mother explaining that she felt:

‘quite sort of proud that I can do this for my child, he has turned into a proper little boy now’ (Tom’s mother).

By setting up routines and support networks parents established and maintained as normal life as possible for their child and family. As things became calmer their child’s care became easier to manage and, for one mother, being at home became was easier than ‘*being in hospital’* (Freya’s mother). However, some parents reported that being at home was not *‘easy’* (Jack’s mother) as they were often challenged not only by their child’s changing needs but also by the need for care support and having to:

‘battle for services and finding that tracheostomy trained person’ (Louise’s mother).

Parents valued and said it was important to draw upon the experiences of other parents whose child had a tracheostomy. They talked of these parents being empathic and able to provide reassurance, noting *‘what we* *want is empathy not sympathy’* (Tom’s father). Overall, in months 4–6, parents described how they took control of the situation and mastered the many transitions and challenges of having a child with a tracheostomy.

### Months 7–12: Feeling more positive and reframing

In months 7–12, parents developed a new sense of themselves as parents of a child who had a tracheostomy. This period was characterised by parents managing to bounce back from challenges, becoming stronger and building a different life to the one that they imagined. Parents described how they reframed their feelings about their child having a tracheostomy, explaining they now saw:

‘a different baby, you don’t even notice it [tracheostomy]’ (Sam’s father).

Seeing their child happy and stable made parents feel more positive and, in retrospect, they realised that the tracheostomy had not been as awful as originally anticipated.

Reframing was evident in the way parents talked of being able to deal with whatever came their way and how they perceived and faced their situation. Parents discussed how their child needing a tracheostomy had ‘*brought life into perspective*’ (Rose’s mother). Reflecting on the past allowed parents to reframe and see how far they had come, and they described feelings of having made it through to the other side. As Jack’s mother explained: *‘I am excited to see how further he goes, I am very hopeful’* (Jack’s mother). Tom’s father talked about the difference in their perspectives as parents 12 months after they found out that Tom needed a tracheostomy. His story is full of hope and gratitude for the life they are living

‘When you think back to what we were doing this time last year. The simple things like the sun being out, the sky is blue. It’s like remember last year when it was sunny we were stuck in the hospital, the sky was grey for us and facing his trachy’ (Tom’s father).

A father also reflected that ‘*it’s unbelievable, what a difference a year makes’* (Tom’s father). Everyday family life that seemed unrealistic at the start became an achievable part of their lives. Parents talked about how their child ‘*actually got to go like a normal child would on her school trip’* (Melody’s mother). One mother explained:

‘we do the everyday stuff, he enjoys being around other kids’ (Tom’s mother).

Reframing played a significant part in how parents perceived and faced their situation, allowing them to move forward with their lives.

## Discussion

This qualitative study aimed to gain insight into the journeys taken by parents, over a 12-month period, whose child needed a tracheostomy. The findings are considered alongside the construct of resilience, to highlight both the risk factors that the parents faced, and resources and cognitions they drew upon to respond to adversity and reframe their beliefs and lives. Put simply, resilience is associated with the ability to bounce back or spring back into shape over time (Simpson, 2005).

This study showed that having a child with a tracheostomy changed the parents’ lives, making it impossible for them to go back to their former state. Parents framed some of their experiences as a struggle which had been emotional and shocking and described feeling stretched. However, their experiences served to strengthen their resolve for the future.

**Figure 1.**
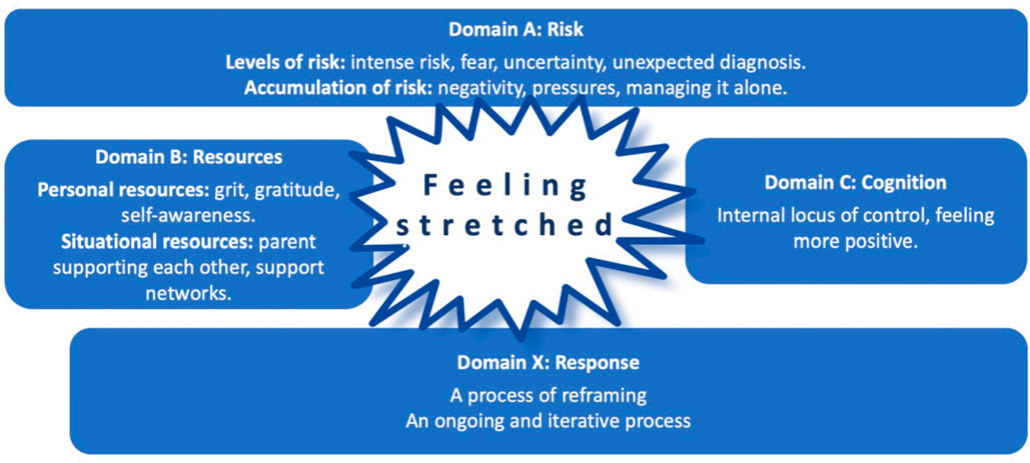
The ABC-X model of parental resilience and reframing.

Resilience is evident in the stories the parents shared. Building from earlier ABC-X model (Ellingsen, et al., 2014), the findings of this study resulted in the development of the ‘ABC-X Model of Parental Resilience and Reframing’. The ABC-X Model of Parental Resilience and Reframing (see Figure 1) draws upon four domains (A, B, C and X) of resilience and acknowledges that throughout the first 12 months of a parent’s journey with a child requiring a tracheostomy, the adversity (risks) they faced can fluctuate as can their responses and their levels of resilience. Domain A focuses on the levels of risks. Domain B focuses on personal resources and situational resources. Domain C focuses on cognitive factors. Domain X focuses on reframing which is the parent’s response to their adversity.

Parents’ resilience was built during times of adversity and required them to draw on their personal (internal) resources such as their determination or grit and self-awareness and their use of situational (external) resources such as support networks (Rutter, 2012; Schofield, 2001). These aspects are typical of Domain B in the model. As seen in other research focussing on parents of children with chronic illness or disability, developing coping strategies (Fairfax et al., 2019) and building resilience is key to helping parents manage adversity and mitigate the stressors they encounter (Delaney, 2022; Lee et al., 2004; Luo et al., 2021). As the parents overcame and reflected on each new experience, it was evident that they became more confident in managing the next challenge. Resilience can be a protective factor for parents under pressure (Bristow et al., 2022; McLoone et al., 2022).

The parents drew upon various personal resources including grit and gratitude in response to the adversities they faced, and these internal resources helped them to accept that their child needed to have a tracheostomy. Grit is emotional strength involving sustained diligent effort in the pursuit of long-term goals with perseverance and passion regardless of adversity (Duckworth, et al., 2007; Stoffel and Cain, 2018; Underdahl et al., 2018). Gratitude is a positive characteristic, an emotion that is important in human relation (McCullough and Tsang, 2004); it promotes a shift in a person’s thinking about what their life lacks to embracing what the good things in their life (Arnout and Almoied, 2021). When grit and gratitude are present together, they can have synergistic benefits (Kleiman et al., 2013) and studies have indicated that positive acknowledgements and gratitude are associated with reframing and growth (Woods et al., 2007). Findings from this study show that by acknowledging that the tracheostomy relieved their child’s breathing difficulties, gratitude meant that parents were able to positively reframe their situation.

Self-awareness played a role, particularly in the early stages of their journey, when despite the threat to their child’s life, they were resistant to the idea of a tracheostomy. However, in the light of seeing their child suffer, parents regulated and modified their negative feelings towards their child’s tracheostomy. Another study that explored self-understanding for parents whose child had a disability came to similar conclusions, noting that self-awareness alongside reflection were crucial factors that strengthened parents’ own values (Haugstvdet et al., 2013).

After the initial periods of uncertainty and anxiety, parents in this study acquired the clinical skills to care for their child and regained a sense of control in their everyday lives; this is reported in other studies (Wilkinson et al., 2020). This internal locus of control is an important aspect of the model (Domain C). Parents took control emotionally and intellectually and mastered the many transitions and challenges of having a child with a tracheostomy; this was an evolving process and it helped them to become resilient and feel less stretched. In this study, as in other studies, the mothers took primary control of their child’s care and tended to be more involved in clinical aspects of care, with the fathers adopting the role of main wage earner (Craig and Bittman, 2008; Craig and Mullan, 2010; Cardinali et al., 2019). However, caring for a child with a chronic condition is a family endeavour and fathers are involved in clinical care (Cardinali et al., 2019). Fathers also experience stress and guilt related to juggling their work and home lives, whilst trying to maintain normality (Hobson and Noyes, 2011). Other studies exploring resilience and an internal locus of control of parents whose children have a disability and cerebral palsy concluded that an internal locus of control can act as a protective mechanism to resilience (Rajan et al., 2018) and may enhance the development of resilience-related qualities (Cohen et al., 2008).

Reframing has been described as a coping strategy which can foster positive growth that, in turn, helps foster resilience (Booth, 2015). Reframing has been reported as the maternal need to ‘cognitively restructure’ a situation that is painful and hard (Krstic et al., 2017: 391] and involves changing the way people see things (Thorpe, 2012). When reframing occurs, both emotional and behavioural changes follow helping people to further manage and resolve the stress (Samios and Baran, 2018). Hope can play a key role in reframing (Cousineau et al., 2019). In this study, reframing (Domain X) created the conditions from which parents bounced back from situations of adversity. As the parents became accustomed to situations that had initially challenged their perceptions and made them feel stretched, they reframed their views, and were able to move on. Inherent within the structure of reframing, was the parents’ belief that they had the resources they needed to make the desired change and redefine their negative situation into a positive one. Other studies of parents of children with chronic illness propose that parents would benefit from a compassionate approach from health professionals (Cousineau et al., 2019), family-focussed practices (Delaney et al., 2022; Lopez-Vargas et al., 2019) and interventions to support and cultivate parental resilience (Delaney et al., 2022; Lopez-Vargas et al., 2019; Luo et al., 2022; Qiu et al., 2021).

Having a child with a tracheostomy changed the parents’ lives. Despite the struggle, shock and emotion they drew on their personal and situational resources, developed a greater sense of control and more positive thoughts (cognitions) about their situation. They reframed their perspectives and built resilience in relation to their child’s care and the challenges arising from their need for a tracheostomy.

### Study limitations

Despite a large catchment area, recruitment was limited to one specialist paediatric hospital, so the parents’ experiences of immediate and ongoing specialist care are not necessarily typical of other settings. More mothers than fathers participated reducing the transferability of paternal perspectives. The inclusion criteria required fluency in English; this criterion means that the findings are not necessarily reflect stories told in different languages and from different cultural perspectives. The positionality of the lead researcher (AF) may have influenced data collection as she had a professional relationship with all participants and despite measures to address an imbalance in the power relationship, this could have impacted on recruitment and data generation. Additionally, as a novice qualitative researcher some opportunities for more in-depth interviews or deeper analytical sights and interpretations may have been missed.

### Implications for practice

This study has highlighted key points, challenges and elements of parents’ journeys when caring for a child requiring and having a tracheostomy. In recognising the timepoints when parents may be especially vulnerable, professionals should be proactive in the provision of information and additional support. The ABC-X Model of Parental Resilience and Reframing offers a useful framework for appreciating parental experiences and the risks, responses and the resources drawn on by parents; it could inform practice by encouraging a focus on supporting capabilities and identifying resources. However, the model needs further investigation across a wider population of parents including those who do not speak English.

## Conclusion

This is the first longitudinal qualitative study to use resilience as a construct to explain the journeys taken by parents whose child needed a tracheostomy. The importance of this research lies in the foundations it lays for new ways of working and supporting parents whose child requires a tracheostomy. There is clear evidence that parents develop resilience over time and the ABC-X Model of Parental Resilience and Reframing shows how this occurs and identifies opportunities for their resilience to be fostered and supported by health professionals.

## References

[bibr1-13674935231169409] Arnout BA Almoied AA (2021) A structural model relating gratitude, resilience, psychological well-being and creativity among psychological counsellors. Counselling and Psychotherapy Research 21(2): 470–488.

[bibr2-13674935231169409] Booth JW (2015) Coping Strategies and Development of Psychological Resilience in Outdoor Education. Canberra, Australia: University Canberra. (Unpublished honours thesis).

[bibr3-13674935231169409] Bristow S Usher K Power T , et al. (2022) Understanding maternal resilience; Lesson learnt from rural mothers caring for a child with a chronic health condition. Journal of Clinical Nursing 31(17–18): 2593–2604. 34693563 10.1111/jocn.16081

[bibr4-13674935231169409] Callans KM Bleiler C Flanagan J , et al. (2016) The transitional experience of family caring for their child with a tracheostomy. Journal of Pediatric Nursing 31(4): 397–403. PMID: 27040188. DOI: 10.1016/j.pedn.2016.02.002. 27040188

[bibr5-13674935231169409] Cardinali P Migliorini L Rania N (2019) The caregiving experiences of fathers and mothers of children with rare diseases in italy: challenges and social support perceptions. Frontiers in Psychology 10: 1780. 31428029 10.3389/fpsyg.2019.01780 PMC6690318

[bibr6-13674935231169409] Chauhan RS (2019) Unstructured interviews: are they really all that bad. Human Resource Development International 25(4): 474–487.

[bibr7-13674935231169409] Cousineau TM Hobbs LM Arthur KC (2019) The role of compassion and mindfulness in building parental resilience when caring for children with chronic conditions: a conceptual model. Frontiers in Psychology 10: 1602. 31428005 10.3389/fpsyg.2019.01602 PMC6690403

[bibr8-13674935231169409] Craig L Bittman M (2008) The incremental time costs of children: an analysis of children’s impact on adult time use in Australia. Feminist Economics 14(2): 59–88. DOI: 10.1080/13545700701880999.

[bibr9-13674935231169409] Craig L Mullan K (2010) Parenthood gender and work-family time in Untied States, Australia, Italy, France and Denmark. Journal of Marriage and Family 72(5): 1344–1361. DOI: 10.1111/j.1741-3737.2010.00769.x.

[bibr10-13674935231169409] Cohen E Biran G Aran A , et al. (2008) Locus of control, perceived parenting style and anxiety in children with Cerebral Palsy. Journal of Developmental and Physical Disabilities 20: 415–423. DOI: 10.1007/s10882-008-9106-8.

[bibr11-13674935231169409] Delaney AE Fu MR McTernan ML , et al. (2022) The associations between resilience and socio-demographic factors in parents who care for their children with congenital heart disease. International Journal of Nursing Sciences 9(3): 321–327. 35891914 10.1016/j.ijnss.2022.06.003 PMC9304995

[bibr12-13674935231169409] Denckla CA Cicchetti D Kubzansky LD , et al. (2020) Psychological resilience: an update on definitions, a critical appraisal, and research recommendations. European Journal of Psychotraumatology 11(1): 1822064. 33244362 10.1080/20008198.2020.1822064 PMC7678676

[bibr13-13674935231169409] Duckworth AL Peterson C Matthews MD , et al. (2007) Grit: perseverance and passion for long-term goals. Journal of Personality and Social Psychology 92(6): 1087–1101. 17547490 10.1037/0022-3514.92.6.1087

[bibr14-13674935231169409] Ellingsen RB Baker L Blacher J , et al. (2014) Resilient parenting of preschool children at developmental risk. Journal of Intellectual Disability 58(7): 664–678. PMID: 23834102 DOI: 10.1111/jir.12063. 23834102

[bibr15-13674935231169409] Fairfax A Brehaut J Colman I , et al. (2019) A systematic review of the association between coping strategies and quality of life among caregivers of children with chronic illness and/or disability. BMC Pediatrics 19(1): 215. 31262261 10.1186/s12887-019-1587-3 PMC6600882

[bibr16-13674935231169409] Flynn A (2018) A Narrative Inquiry into the Stories Parents Tell of Having a Child with a Tracheostomy. PhD Thesis. University of Central Lancashire. Available at: https://clok.uclan.ac.uk/29253/1/29253FlynnAlisonFinale-Thesis%28MasterCopy%29.pdf.

[bibr17-13674935231169409] Flynn AP Carter B Bray L , et al. (2013) Parents’ experiences and views of caring for a child with a tracheostomy; a literature review. International Journal of Pediatric Otorhinolaryngology 77(10): 1630–1634. PMID: 23953483 DOI: 10.1016/j.ijporl.2013.07.020. 23953483

[bibr18-13674935231169409] Frank AW (2010) Letting Stories Breathe: A Socio-Narratology. London: The University of Chicago Press.

[bibr19-13674935231169409] Hartnick CJ Bissell C Parsons SK (2003) The impact of pediatric tracheotomy on parental caregiver burden and health status. Archives of Otolaryngology-Head and Neck Surgery 129: 1065–1069. PMID: 14568788 doi:. DOI: 10.1001/archotol.129.10.1065. 14568788

[bibr20-13674935231169409] Haugstvedt KTS Graff-Iversen S Bukholm IRK , et al. (2013). Processes of enhanced self-understanding during a counselling programme for parents of children with disabilities. Scandinavian Journal of Caring Sciences, 27, 108–116. PMID: 22620983 DOI: 10.1111/j.1471-6712.2012.01008.x. 22620983

[bibr21-13674935231169409] Hill R (1949) Families under Stress. New York: Harper & Row.

[bibr22-13674935231169409] Hobson L Noyes J (2011) Fatherhood and children with complex health care needs; qualitative study of fathering, caring and parenting. BMC Nursing 10: 5. 21496238 10.1186/1472-6955-10-5 PMC3094306

[bibr23-13674935231169409] Kleiman EM Adams LM Kashdan TB , et al. (2013) Gratitude and grit indirectly reduce risk of suicidal ideations by enhancing meaning in life; evidence for a mediated moderation model. Journal of Research in Personality 47: 539–546. DOI: 10.1016/j.jrp.2013.04.007.

[bibr24-13674935231169409] Krstic T Mihic L Oros M (2017) Coping strategies and resolutions in mothers of children with cerebral palsy. Journal of Loss and Trauma 22(5): 385–395. DOI: 10.1080/15325024.2017.1297659.

[bibr25-13674935231169409] Lee I Lee EO Kim HS , et al. (2004) Concept development of family resilience: A study of Korean families with a chronically ill child. Journal of Clinical Nursing 13: 636–645. PMID: 15189417. DOI: 10.1111/j.1365-2702.2004.00845.x. 15189417

[bibr27-13674935231169409] Lopez-Vargas P Tong A Crowe S , et al. (2019) Research priorities for childhood chronic conditions: a workshop report. Archives of Disease in Childhood 104: 237–245. 30279157 10.1136/archdischild-2018-315628

[bibr28-13674935231169409] Luo D Gu W Bao Y , et al. (2021) Resilience outstrips the negative effect of caregiver burden on quality of life among parents of children with type 1 diabetes: an Application of Johnson-Neyman Analysis. Journal of Clinical Nursing 30(13–14): 1884–1892. 33656212 10.1111/jocn.15739

[bibr29-13674935231169409] Luo Y Li HCW Xia W , et al. (2022) The lived experience of resilience in parents of children with cancer: a phenomenological study. Frontiers in Pediatrics 10: 871435. 35707743 10.3389/fped.2022.871435 PMC9189362

[bibr30-13674935231169409] Masten AS (2001) Ordinary magic: Resilience processes in development. The American Psychologist 56(3): 227–238. DOI: 10.1037/0003-066X.56.3.227. 11315249

[bibr31-13674935231169409] Montagnino BA Mauricio RV (2004) The child with a tracheostomy and gastrostomy: parental stress and coping in the home-a pilot study. Pediatric Nursing 30(5): 373–380, 401. PMID 15587530. 15587530

[bibr32-13674935231169409] McCann D Bull R Winzenberg T (2015) Sleep deprivation in parents caring for children with complex needs at home: a mixed methods systematic review. Journal of Family Nursing 21(1): 86–118. PMID 25527511. DOI: 10.1177/1074840714562026. 25527511

[bibr33-13674935231169409] McCubbin HI Patterson JM (1983) The family stress process: the double ABCX model of adjustment and adaptation. Marriage and Family Review 6(1–2): 7–37. DOI: 10.1300/J002v06n01_02.

[bibr34-13674935231169409] McCullough ME Tsang J (2004) Parent of the virtues? The prosocial contours of gratitude. In: Emmons RA McCullough ME (eds), The Ppsychology of Gratitude. New York, NY: Oxford University Press, pp. 123–141.

[bibr35-13674935231169409] McDonald J McKinlay E Keeling S , et al. (2016) Becoming an expert carer: the process of family carers learning to manage technical health procedures at home. Journal of Advanced Nursing 72(9): 2173–2184. PMID: 27113636. DOI: 10.1111/jan.12984. 27113636

[bibr36-13674935231169409] McLoone J Wakefield CE Marshall GM , et al. (2022) It's made a really hard situation even more difficult: The impact of COVID-19 on families of children with chronic illness. PLoS One 17(9): e0273622. 36048846 10.1371/journal.pone.0273622 PMC9436103

[bibr37-13674935231169409] McNamara DG Dickinson AR Byrnes CA (2009) The perceptions and preferences of parents of children with tracheostomies in a study of humidification therapy. Journal of Child Health Care: For Professionals Working with Children in the Hospital and Community 13(3): 179–197. DOI: 10.1177/1367493509336686. 19713403

[bibr38-13674935231169409] Noyes J (2014) The concept of resilience in Children’s Health and Social Care policy. In: DeMichelis C Ferrari M (eds), Child and Adolescent Resilience Within Medical Contexts. Integrating Research and Practice. Switzerland: Springer.

[bibr58-13674935231169409] Patterson JM (1988) Families experiencing stress: I. The Family Adjustment and Adaptation Response Model: II. Applying the FAAR Model to health-related issues for intervention and research. Family Systems Medicine 6(2): 202–237. DOI: 10.1037/h0089739.

[bibr39-13674935231169409] Qiu Y Xu L Pan Y , et al. (2021) Family Resilience, Parenting Styles and Psychosocial Adjustment of Children With Chronic Illness: A Cross-Sectional Study. Frontiers in Psychiatry 12: 646421. 34054605 10.3389/fpsyt.2021.646421 PMC8149598

[bibr40-13674935231169409] Rajan AM Srikrishna G Romate J (2018) Resilience and locus of control of parents having a child with intellectual disability. Journal of Developmental and Physical Disabilities 30: 297–306. DOI: 10.1007/s10882-018-9586-0.

[bibr41-13674935231169409] Riessman CK (2008) Narrative Methods for the Human Sciences. CA: Sage.

[bibr42-13674935231169409] Rutter M (2012) Resilience as a dynamic concept. Development and Psychopathology, 24(2):335–344. PMID: 22559117 DOI: 10.1017/S0954579412000028. 22559117

[bibr43-13674935231169409] Samios C Baran S (2018) Couple adjustment to a stressful life event: a dyadic investigation of the roles of positive reframing and perceived benefits. Anxiety, Stress and Coping 31(I2): 6–19.DOI: 10.1080/10615806.2017.1420173. 29272954

[bibr44-13674935231169409] Schofield G (2001) Resilience and family placement: a lifespan perspective. Adoption and Fostering 25(6). DOI: 10.1177/2F030857590102500303.

[bibr45-13674935231169409] Simpson J (2005) Oxford English Dictionary. 3rd edition. New York NY: Oxford University Press.

[bibr46-13674935231169409] Stoffel JM Cain J (2018) Review of Grit and Resilience Literature within Health Professions Education. American Journal of Pharmaceutical Education 82(2): 6150. 29606705 10.5688/ajpe6150 PMC5869747

[bibr47-13674935231169409] Southwick SM Bonanno GA Masten AS , et al. (2014) Resilience definitions, theory, and challenges: interdisciplinary perspectives. European Journal of Psychotraumatology 5. 10.3402/ejpt.v5.25338 PMC4185134 25317257

[bibr48-13674935231169409] Thorpe M (2012) Reframing our ways of working and stretching the creative spirit. Design Management Review 23(3): 78–85.

[bibr50-13674935231169409] Underdahl L Jones-Meineke T Duthely LM (2018) Reframing physician engagement: An analysis of physician resilience, grit, and retention. International Journal of Healthcare Management 11(3): 243–250.

[bibr51-13674935231169409] Ungar M (2019) Designing resilience research: Using multiple methods to investigate risk exposure, promotive and protective processes, and contextually relevant outcomes for children and youth. Child Abuse and Neglect 96: 104098. 31376582 10.1016/j.chiabu.2019.104098

[bibr52-13674935231169409] Watters KF (2017) Tracheostomy in Infants and Children. Respiratory Care 62(6): 799–825. DOI: 10.4187/respcare.05366. 28546379

[bibr53-13674935231169409] Wilkinson C Bray L Carter B , et al. (2020) Not a nurse but more than a mother: the everyday geographies of mothering children with complex heath care needs. Children's Geographies 19: 158–171. DOI: 10.1080/14733285.2020.1755420.

[bibr54-13674935231169409] Windle G (2011) What is resilience? A review and concept analysis. Reviews in Clinical Gerontology 21: 152–169. DOI: 10.1017/S095925981000042.

[bibr55-13674935231169409] Woods AM Joseph S Linley PA (2007) Coping style as a psychological resource of grateful people. Journal of Social and Clinical Psychology 26: 1076–1093. DOI: 10.1521/jscp.2007.26.9.1076.

[bibr57-13674935231169409] Ziebland S (2013) Narrative Interviewing. In: Zielband S Coulter A Calabrese JD , et al. (eds), Understanding and Using Health Experiences. Improving Patient Care. Oxford: Oxford University Press.

